# Demethylation of Circulating Estrogen Receptor Alpha Gene in Cerebral Ischemic Stroke

**DOI:** 10.1371/journal.pone.0139608

**Published:** 2015-09-30

**Authors:** Hsiu-Fen Lin, Edward Hsi, Yi-Chu Liao, Brian Chhor, Jessica Hung, Suh-Hang H. Juo, Ruey-Tay Lin

**Affiliations:** 1 Department of Neurology, Kaohsiung Medical University Hospital, Kaohsiung, Taiwan; 2 Department of Neurology, College of Medicine, Kaohsiung Medical University, Kaohsiung, Taiwan; 3 Department of Medical Research, Kaohsiung Medical University Hospital, Kaohsiung, Taiwan; 4 Department of Medical Genetics, College of Medicine, Kaohsiung Medical University, Kaohsiung, Taiwan; 5 Department of Neurology, Taipei Veterans General Hospital, Taipei, Taiwan; 6 Department of Neurology, National Yang-Ming University School of Medicine, Taipei, Taiwan; 7 Department of Human Biology, Stanford University, Stanford, California, United States of America; 8 Department of Bioengineering, University of Washington, Seattle, United States of America; Università di Napoli Federico II, ITALY

## Abstract

**Background:**

Estrogen is involved in neuron plasticity and can promote neuronal survival in stroke. Its actions are mostly exerted via estrogen receptor alpha (ERα). Previous animal studies have shown that ERα is upregulated by DNA demethylation following ischemic injury. This study investigated the methylation levels in the ERα promoter in the peripheral blood of ischemic stroke patients.

**Methods:**

The study included 201 ischemic stroke patients, and 217 age- and sex-comparable healthy controls. The quantitative methylation level in the 14 CpG sites of the ERα promoter was measured by pyrosequencing in each participant. Multivariate regression model was used to adjust for stroke traditional risk factors. Stroke subtypes and sex-specific analysis were also conducted.

**Results:**

The results demonstrated that the stroke cases had a lower ERα methylation level than controls in all 14 CpG sites, and site13 and site14 had significant adjusted p-values of 0.035 and 0.026, respectively. Stroke subtypes analysis showed that large-artery atherosclerosis and cardio-embolic subtypes had significantly lower methylation levels than the healthy controls at CpG site5, site9, site12, site13 and site14 with adjusted *p* = 0.039, 0.009, 0.025, 0.046 and 0.027 respectively. However, the methylation level for the patients with small vessel subtype was not significant. We combined the methylation data from the above five sites for further sex-specific analysis. The results showed that the significant association only existed in women (adjusted *p* = 0.011), but not in men (adjusted *p* = 0.300).

**Conclusions:**

Female stroke cases have lower ERα methylation levels than those in the controls, especially in large-artery and cardio-embolic stroke subtypes. The study implies that women suffering from ischemic stroke of specific subtype may undergo different protective mechanisms to reduce the brain injury.

## Introduction

Cerebral ischemic stroke is a leading cause of long-term disability worldwide. After ischemia, the neuron cells may activate multiple death cascades, including apoptosis, necrosis, and autophagy. However, several neuroprotective mechanisms have been identified to reduce neuronal injury and alleviate the insult after ischemic stress [1,2]. Estrogen is one of them to promote neuronal survival and reduce disability in patients with ischemic stroke [3,4].

In addition to acting as a “sex hormone”, estrogen has been documented to provide a multifaceted modulation of neurons, including plasticity and neuroprotection in stroke [3,4]. Most of estrogen’s actions are exerted via nuclear receptors and estrogen receptor alpha (ERα) is the most important one for estrogen-mediated neuroprotection following focal cerebral ischemia [5]. In experimental stroke models, the estrogen protective effect is lost in the ERα knockout mice [6]. Additionally, mRNA and protein levels of ERα are significantly increased in the cortex of middle cerebral artery occlusion (MCAO) mice [7]. These findings suggest that the ERα is a critical link in mediating the protective effects of estrogen in ischemic brain injury.

DNA methylation is a major type of epigenetic regulation that affects disease pathogenesis. In adult non-gamete cells, DNA methylation typically takes place in a cytosine–guanine (CpG) dinucleotide context, also known as a CpG site. In general, DNA methylation will silence gene expression. The DNA methylation status can be altered by environmental changes [8]. Accumulating evidence has indicated that an increased methylation level in the ERα promoter region is negatively associated with ER expression in several diseases, including breast cancer, prostate cancer, and atherosclerosis [9–11]. The methylation of the ERα gene involved in ischemic stroke has drawn attention in a rodent stroke study recently [12]. Westberry et al have found that the methylation level of ERα decreased following MCAO induction in rats. Interestingly, brain ischemic that leads to demethylation in the ERα promoter only occurs in female rats, but not in male rats [12]. However, the effect of methylation levels in the ERα promoter region has not yet to be explored among ischemic stroke patients.

This study aims to investigate the level of ERα methylation in the peripheral blood of ischemic stroke patients. As the disease severity and outcome varied among stroke subtypes [13], we also examined the methylation status of the ERα gene in different stroke subtypes. Since gender might have an influence on the ERα genetic predisposition, we assessed the methylation level of ERα between male and female patients.

## Materials and Methods

### Subjects

#### Stroke subjects

Patients of ischemic stroke with ages between 40 and 80 years old were selected from our existing patient cohort, all of whom enrolled from the Kaohsiung Medical University Hospital and the Taichung Veterans General Hospital in Taiwan [14]. These cases were selected using the following criteria: (1) stroke subjects in our existing cohort were selected only when the first stroke event occurred before age 80, (2) stroke cases were divided into four age groups (40–50 y/o, 51–60 y/o, 61–70 y/o, 71–80 y/o), and (3) equal number of male and female stroke patients were selected from each age group randomly. The available DNA samples were checked from the selected patients and the final 201 patients were included in this study. All patients had a standard stroke investigation that included laboratory examination and cranial computed tomography (CT) or magnetic resonance imaging (MRI). Ischemic stroke status was established when the brain imaging revealed acute infarction and showed no evidence of hemorrhage. Stroke subtypes were classified based on the Trial of ORG 10172 in Acute Stroke Treatment (TOAST) [15].

#### Control subjects

The healthy controls were selected from the subjects of self-reported stroke- and myocardial infarction (MI)-free volunteers recruited at the Kaohsiung Medical University Hospital through an advertisement soliciting volunteers [16]. Two hundred and seventeen age- and sex-comparable controls were selected by a table of random numbers. To minimize the age effect, we tried to select an equal number of cases and controls from each age group. We performed the above procedure for men and women separately to minimize gender confounds. The random number tables were generated by the SPSS statistical software.

For each participant, socio-demographic information and medical history of hypertension, diabetes, hyperlipidemia, and cigarette smoking were obtained. Total cholesterol, triglycerides, and glucose levels were measured from venous blood after fasting for at least 8 hours. Hypertension was defined as systolic or diastolic blood pressure ≥140/90 mm Hg or anti-hypertensive medication use. Diabetes was defined as fasting blood glucose ≥126 mg/dl or known treatment for diabetes. Hypercholesterolemia was defined as serum levels of total cholesterol ≥200 mg/dl or use of lipid lowering medication. The Kaohsiung Medical University Hospital and the Taichung Veterans General Hospital Institutional Review Boards approved the study and every participant provided written informed consent.

### Genomic DNA extraction and detection of ERα methylation

Genomic DNA was isolated using a commercially available DNA extraction kit (Gentra; Qiagen, Hilden, Germany). DNA sample was then treated with sodium bisulfate, converting unmethylated cytosine (C) to uracil and leaving methylated C intact, by using an EpiTect Fast Bisulfite Kit (Qiagen) following the manufacturer’s recommendations. The completion of bisulfite treatment was assayed by detecting unconverted bisulfite cytosine outside the CpG based on the assumption of non-CpG cytosines were mainly unmethylated. The method for the assessment is as follow: the pyrosequencer PyroMark Q24 has a feature that acts as quality control for complete bisulfite conversion of DNA. When the assay encounters a unmethylated C not followed by a guanine (G), that C should be fully converted to thymine (T) after bisulfite treatment and PCR, if the bisulfate treatment upfront was successful. Subsequently, it should be presented in the pyrogram as T = 100%. On the other hand, if the non-CpG cytosine is methylated, the methylated C will not be eventually converted to T. Accordingly, the pyrogram from the assay will not yield a perfect conversation to T and instead a “Failed” quality assessment will be assigned by the Q24 software. This acts as a useful quality control for full conversion of unmethylated C residues during bisulfite treatment and PCR. We assessed the completion of bisulfite treatment at all eight non-CpG cytosines in our sequence region in 10 randomly selected samples. All non-CpG cytosine showed fully bisulfite conversion.

**Fig 1:**
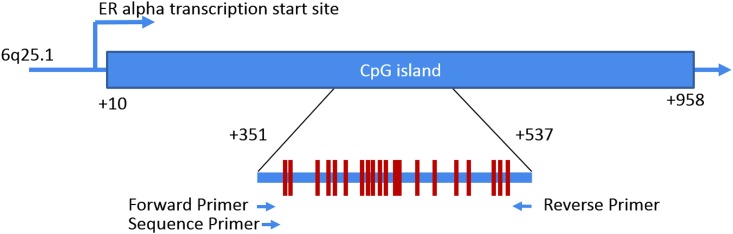
Schematic diagram of the distribution of CpGs in estrogen receptor α promoter CpG island.

The original ERα methylation assay was designed by PyroMark Assay Design 2.0 software (Qiagen) to cover 20 CpG sites in the CpG island of the ERα promoter. The bisulfite-modified DNA was used to amplify the 187-bp product in the promoter of ERα gene (Fig 1) (primers are shown in S1 Table). The quantitative ERα methylation level at the CpG sites was evaluated via pyrosequencing (PyroMark Q24;Qiagen). Universal unmethylated and methylated DNAs were run as controls. Methylation quality check and quantification were performed using the PyroMark Q24 2.0.6 software (Qiagen). The level of ERα methylation was quantified as percentage of methylated cytosine within the sum of methylated and unmethylated cytosines.

### Statistical analysis

Student’s t-test and chi-square test were used to analyze the characteristics of study subjects. Analysis for an association between the ERα methylation level and the phenotypes of interest (stroke as a whole and stroke subtypes) was first performed using the Student’s t-test. Multivariate logistic regression analysis was conducted to adjust for sex, age, smoking, hypertension, diabetes, and hypercholesterolemia. We also analyzed the sex-specific effect, given that ERα methylation level was only changed in female rats [12]. The SPSS 18.0 version for Windows (SPSS Inc., Chicago, Illinois, USA) was used for statistical analysis. A two-sided p-value less than 0.05 was considered statistically significant.

## Results

The demographic characteristics of the study subjects are presented in Table 1. Since the cases and controls were selected to be age- and sex-comparable, these two factors were no longer significantly different. Nevertheless, traditional risk factors such as the prevalence of hypertension, diabetes, hypercholesterolemia, and smoking were significantly higher in stroke cases than in controls, as expected. For stroke subtypes, there were 39 (19.4%) cases of large-artery atherosclerosis, 20 (10.0%) of cardio-embolism, 89 (44.3%) of small vessel occlusion, and 53 (26.4%) of undetermined etiology based on the TOAST classification [15].

**Table 1:** Demographic characteristics of the study participants.

	Stroke, n = 201	Controls, n = 217	*p* value
Age (yr)	60.8±10.3	61.4±11.0	0.554
Male (%)	101 (50.2)	101 (46.5)	0.449
Hypertension (%)	154 (76.6)	99 (45.6)	<0.001
Diabetes (%)	91 (45.3)	32 (14.7)	<0.001
Hypercholesterolemia (%)	102 (50.7)	59 (27.2)	<0.001
Current& ever smoker (%)	69 (34.3)	34 (15.7)	<0.001
Stroke subtype			
Large-artery atherosclerotic	39 (19.4)		
Cardio-embolism	20 (10.0)		
Small vessel	89 (44.3)		
Undetermined	53 (26.4)		

Data are shown as mean± SD for quantitative variables and n (%) for qualitative variables

The length of our target sequence (187-bp) is longer than the suggested length (within 80–100 bp) by the manufacture, which is due to limited choices to design primers in the CG rich region. The results from pyrosequencing indicated that six sites (sites15–20) had low quality of sequence data that were excluded for further analysis. Therefore, the present study only presented data at 14 CpG sites. The methylation levels in the peripheral blood at 14 ERα promoter CpG sites in stroke cases and healthy controls are shown in Table 2. In general, ERα methylation levels were lower among the ischemic stroke cases compared to those in the controls. The p-values at CpG site13 and site14 were significant (adjusted *p* = 0.035 and 0.026, respectively).

**Table 2:** Association between ischemic stroke and estrogen receptor α promoter methylation level.

	Controls, n = 217	Stroke, n = 201		
CpG position/ Methylation %	Mean±SD	Mean ± SD	Crude *p* value	Adjusted *p* value
Site1	5.08±2.02	4.71±2.24	0.078	0.353
Site2	3.93±1.62	3.60±1.63	**0.043**	0.284
Site3	5.24±2.64	4.90±3.01	0.224	0.512
Site4	3.89±1.44	3.64±1.51	0.079	0.500
Site5	4.34±2.10	3.88±2.01	**0.021**	0.221
Site6	3.71±1.41	3.32±1.69	**0.012**	0.111
Site7	4.95±1.75	4.61±2.13	0.073	0.385
Site8	3.65±1.57	3.35±2.40	0.136	0.287
Site9	2.93±1.10	2.64±1.47	**0.023**	0.083
Site10	4.90±1.70	4.60±2.43	0.142	0.854
Site11	4.76±2.21	4.27±2.05	**0.018**	0.078
Site12	4.40±1.74	4.02±2.70	0.097	0.079
Site13	3.05±1.60	2.60±1.13	**0.001**	**0.035**
Site14	7.87±3.62	6.95±2.58	**0.003**	**0.026**

Adjusted p value was adjusted for age, sex, hypertension, diabetes, hypercholesterolemia, and smoking

There are differences in stroke severity and outcomes between stroke subtypes [13,17]. Large-artery atherosclerosis and cardio-embolism are associated with a more adverse outcome and early stroke recurrence. Small vessel occlusion, on the contrary, is associated with the lowest stroke severity and mortality [13,17]. Therefore, large-artery atherosclerosis and cardio-embolism were combined as one group (LAA/CE group) and small vessel occlusion was treated as the other group (SVO group) while we evaluated the ERα methylation status in stroke subtypes. Compared with the healthy controls, patients in the LAA/CE group had lower ERα methylation levels at all CpG positions, especially at CpG site5, site9, site12, site13 and site14 with adjusted *p* = 0.039, 0.009, 0.025, 0.046 and 0.027 respectively (Table 3). However, no significant difference in methylation level was observed between patients in the SVO group and the healthy controls in any of the ERα CpG sites.

**Table 3:** Stratification analysis for association between ischemic stroke subtypes and estrogen receptor α promoter methylation.

	Control	Small vessel		Large-artery atherosclerosis and cardio-embolic	
CpG position/Methylation%	n = 217	n = 89	Adjusted *p* value	n = 59	Adjusted *p* value
Site1	5.08±2.02	4.79±2.18	0.618	4.57±2.54	0.458
Site2	3.93±1.62	3.79±1.79	0.908	3.43±1.62	0.339
Site3	5.24±2.64	5.18±3.36	0.948	4.78±3.03	0.724
Site4	3.89±1.44	3.81±1.64	0.745	3.40±1.34	0.145
Site5	4.34±2.10	3.99±1.95	0.414	3.55±1.58	**0.039**
Site6	3.71±1.41	3.40±1.70	0.272	3.29±2.04	0.307
Site7	4.95±1.75	4.59±1.81	0.308	4.47±2.09	0.229
Site8	3.65±1.57	3.39±2.03	0.373	3.37±3.30	0.485
Site9	2.93±1.10	2.63±1.10	0.125	2.40±1.05	**0.009**
Site10	4.90±1.70	4.65±2.66	0.952	4.60±2.48	0.841
Site11	4.76±2.21	4.36±1.72	0.294	4.27±2.91	0.342
Site12	4.40±1.74	4.09±2.18	0.400	3.60±1.94	**0.025**
Site13	3.05±1.60	2.70±1.21	0.366	2.48±1.13	**0.046**
Site14	7.87±3.62	7.03±2.51	0.211	6.68±2.48	**0.027**

Adjusted p value was adjusted for age, sex, hypertension, diabetes, hypercholesterolemia, and smoking

**Fig 2:**
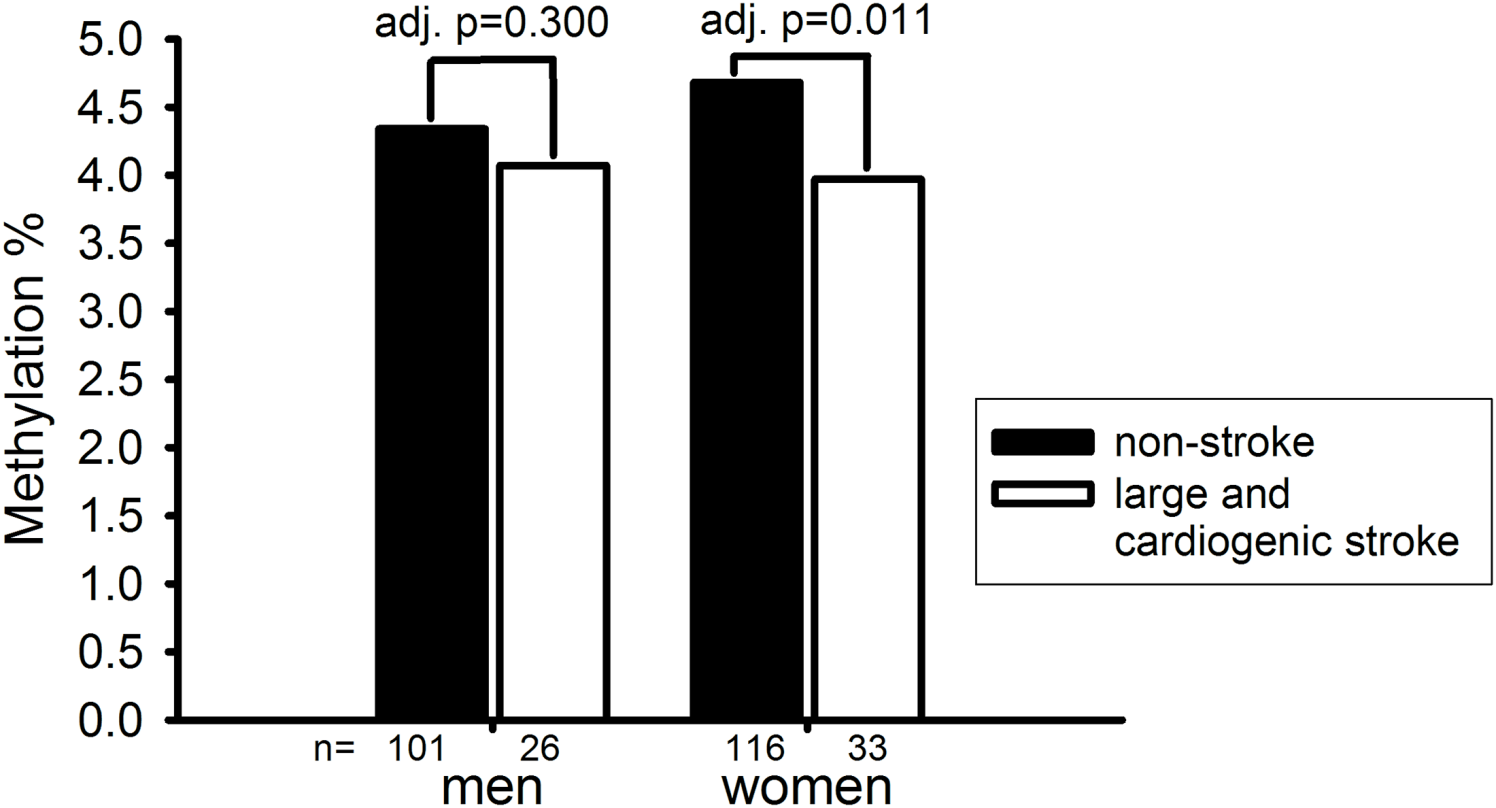
Sex-specific analysis for the association between estrogen receptor α promoter methylation level and large-artery atherosclerosis and cardio-embolic stroke subtypes.

Since demethylation of the ERα gene following ischemic stroke was shown in female but not male rats [12], we further tested the sex-specific effect of ERα methylation status in the LAA/CE group. The methylation levels of five significant CpG sites shown in Table 3 (i.e. CpG site5, site9, site12, site13 and site14) were different between male and female patients (S2 Table). The average methylation levels of these five significant CpG sites were used for subsequent analysis. Compared with female controls, female cases had a significant lower methylation level in the ERα promoter (3.97% vs 4.68%, adjusted *p* = 0.011) (Fig 2). Although male cases also had a lower ERα methylation level compared with male controls, the difference was not statistically significant (4.07% vs 4.34%, adjusted *p* = 0.300).

## Discussion

The present study demonstrates that in human data, female stroke cases have lower ERα methylation levels than those in the controls, which is consistent with the previous experimental findings in stroke animals [12]. However, the association was primarily in the patients of LAA/CE stroke subtypes. While the patients of SVO stroke also had a lower methylation level than the control subjects, the difference did not yield statistical significance. The data imply that women suffering from a major ischemic stroke may cause a more significant change in ERα methylation levels to reduce the brain injury. This finding also suggests that treatments for ischemic stroke may vary between female and male patients. In addition, the beneficial effect of estrogen therapy in acute ischemic stroke [18] may require further analysis in sex-specific and stroke subtype-specific manners.

DNA methylation is a well characterized epigenetic change that contributes to transcriptional regulation [8]. Although aberrant DNA methylation was extensively explored in cancer studies, the importance of DNA methylation has been increasingly recognized in atherosclerosis [19,20]. Only a few human studies have addressed the correlation between DNA methylation level and stroke [21–23]. Both Baccarelli et al.[21] and our group [23] found lower global methylation levels in Long Interspersed Nucleotide Element 1 (LINE-1) in the male stroke cases than controls. A higher methylation level in the promoter of the brain derived neurotrophic factor (BDNF) was reported to be associated with a poor outcome one year after stroke [22]. To our knowledge, this is the first human stroke study to show the ERα promoter methylation data that is consistent to the previous rodent stroke study [12].

The crucial role of estrogen in neuroprotection following stroke has been reported in experimental stroke models [24,25]. It is known that the effects of estrogen on the brain following ischemic injury are largely related to regional expression of ERα [7]. It is also suggested that DNA methylation is a major mechanism for ERα upregulation following injury [12]. The ERα promoter is highly methylated in the adult rodent cortex [26], but the methylation level decreases dramatically and rapidly following a neuronal ischemic insult [12], which may account for the protective effect of estrogen in the ischemia models. Some drugs have been shown to be associated with the changes of DNA methylation *in vitro and in vivo* [27,28]. Our laboratory recently reported that a traditional Chinese medicine can increase microRNA-152 expression, which leads to a reduction of DNA methylation in the ERα promoter [20]. In addition to direct use of estrogen treatment, medications that can affect ERα methylation may be clinically useful for stroke treatment.

The increased ERα expression in stroke mice occurs in neurons but not in astrocytes or microglial cells [7]. Stroke caused by LAA/CE subtypes generally involves a large area of cerebral cortex, whereas SVO stroke subtype involves deep cerebral white matter [29]. These may explain why the present study shows the significant results in the LAA/CE subtypes, but not in the SVO subtype.

The gender may have an influence not only on stroke risk but also on stroke outcomes [30–32]. Recently, Tian et al. found that female and male stroke patients had different gene expression patterns in the blood [33]. Although it is still unclear whether alterations in gene expression or epigenetic modifications in the blood of stroke patients can reflect a similar change in the brain, these data provide evidence of gender-specific changes in the DNA and RNA levels after ischemic stroke.

This study has some limitations. The current study used cross-section data and therefore the observed association might not be a causal relationship. However, our result is consistent with the report from the brain tissue of a rodent stroke study [12]. We explored the relationship between ischemic stroke and ERα methylation level in the genomic DNA isolated from peripheral blood rather than brain tissue. Although it may be hard to validate the correlation of methylation pattern between brain and peripheral blood, blood sample has its medical value because of easy access and better practical implications. We took the generally accepted assumption that DNA methylation almost exclusively occurred in CpG dinucleotides in mammals. Our PCR primer design was based on this assumption. However, non-CpG methylation had been reported [34]. The same group recently showed that PCR primer based on such an assumption might cause the underestimation of high DNA methylation [35]. Therefore, our approach might also underestimate the significant level if our PCR primer covered methylated non-CpG cytosines.

This study demonstrates that female patients with ischemic stroke have lower ERα methylation level. This association is especially distinctive among patients with the large vessel and embolic stroke subtypes. The results suggest that elevated ERα due to the decrease of ERα methylation level in female stroke patients may serve as a natural protective mechanism to prevent further neuronal damage.

## Supporting Information

S1 Table Primers for Estrogen receptor 1(ESR1) methylation. (DOCX)

S2 Table Stratification analysis for estrogen receptor α promoter methylation status in large-artery atherosclerosis and cardio-embolic stroke subtypes by sex. (DOCX)

## References

[1] Marsh BJ, Williams-Karnesky RL, Stenzel-Poore MP (2009) Toll-like receptor signaling in endogenous neuroprotection and stroke. Neuroscience 158: 1007–1020. 10.1016/j.neuroscience.2008.07.067 18809468 PMC2674023

[2] Trendelenburg G, Dirnagl U (2005) Neuroprotective role of astrocytes in cerebral ischemia: focus on ischemic preconditioning. Glia 50: 307–320. 15846804 10.1002/glia.20204

[3] Simpkins JW, Rajakumar G, Zhang YQ, Simpkins CE, Greenwald D, Yu CJ, et al (1997) Estrogens may reduce mortality and ischemic damage caused by middle cerebral artery occlusion in the female rat. J Neurosurg 87: 724–730. 9347981 10.3171/jns.1997.87.5.0724

[4] Toung TJ, Traystman RJ, Hurn PD (1998) Estrogen-mediated neuroprotection after experimental stroke in male rats. Stroke 29: 1666–1670. 9707210 10.1161/01.str.29.8.1666

[5] Schreihofer DA, Ma Y (2013) Estrogen receptors and ischemic neuroprotection: who, what, where, and when? Brain Res 1514: 107–122. 10.1016/j.brainres.2013.02.051 23500634

[6] Dubal DB, Zhu H, Yu J, Rau SW, Shughrue PJ, Merchenthaler I, et al (2001) Estrogen receptor alpha, not beta, is a critical link in estradiol-mediated protection against brain injury. Proc Natl Acad Sci U S A 98: 1952–1957. 11172057 10.1073/pnas.041483198 PMC29363

[7] Dubal DB, Rau SW, Shughrue PJ, Zhu H, Yu J, Cashion AB, et al (2006) Differential modulation of estrogen receptors (ERs) in ischemic brain injury: a role for ERalpha in estradiol-mediated protection against delayed cell death. Endocrinology 147: 3076–3084. 16527848 10.1210/en.2005-1177

[8] Bird A (2002) DNA methylation patterns and epigenetic memory. Genes Dev 16: 6–21. 11782440 10.1101/gad.947102

[9] Li LC, Chui R, Nakajima K, Oh BR, Au HC, Dahiya R. (2000) Frequent methylation of estrogen receptor in prostate cancer: correlation with tumor progression. Cancer Res 60: 702–706. 10676656

[10] Post WS, Goldschmidt-Clermont PJ, Wilhide CC, Heldman AW, Sussman MS, Ouyang P, et al (1999) Methylation of the estrogen receptor gene is associated with aging and atherosclerosis in the cardiovascular system. Cardiovasc Res 43: 985–991. 10615426 10.1016/s0008-6363(99)00153-4

[11] Yoshida T, Eguchi H, Nakachi K, Tanimoto K, Higashi Y, Suemasu K, et al (2000) Distinct mechanisms of loss of estrogen receptor alpha gene expression in human breast cancer: methylation of the gene and alteration of trans-acting factors. Carcinogenesis 21: 2193–2201. 11133808 10.1093/carcin/21.12.2193

[12] Westberry JM, Prewitt AK, Wilson ME (2008) Epigenetic regulation of the estrogen receptor alpha promoter in the cerebral cortex following ischemia in male and female rats. Neuroscience 152: 982–989. 10.1016/j.neuroscience.2008.01.048 18353557 PMC2515597

[13] Lavados PM, Sacks C, Prina L, Escobar A, Tossi C, Araya F, et al (2007) Incidence, case-fatality rate, and prognosis of ischaemic stroke subtypes in a predominantly Hispanic-Mestizo population in Iquique, Chile (PISCIS project): a community-based incidence study. Lancet Neurol 6: 140–148. 17239801 10.1016/S1474-4422(06)70684-6

[14] Lin HF, Tsai PC, Liao YC, Lin TH, Tai CT, Juo SH, et al (2011) Chromosome 9p21 genetic variants are associated with myocardial infarction but not with ischemic stroke in a Taiwanese population. J Investig Med 59: 926–930. 10.231/JIM.0b013e318214ea49 21415773

[15] Adams HP Jr., Bendixen BH, Kappelle LJ, Biller J, Love BB, Gordon DJ, et al (1993) Classification of subtype of acute ischemic stroke. Definitions for use in a multicenter clinical trial. TOAST. Trial of Org 10172 in Acute Stroke Treatment. Stroke 24: 35–41. 7678184 10.1161/01.str.24.1.35

[16] Lin HF, Liu CK, Liao YC, Lin RT, Chen CS, Juo SH. (2010) The risk of the metabolic syndrome on carotid thickness and stiffness: sex and age specific effects. Atherosclerosis 210: 155–159. 10.1016/j.atherosclerosis.2009.11.027 20035939

[17] Grau AJ, Weimar C, Buggle F, Heinrich A, Goertler M, Neumaier S, et al (2001) Risk factors, outcome, and treatment in subtypes of ischemic stroke: the German stroke data bank. Stroke 32: 2559–2566. 11692017 10.1161/hs1101.098524

[18] Liu R, Liu Q, He S, Simpkins JW, Yang SH (2010) Combination therapy of 17beta-estradiol and recombinant tissue plasminogen activator for experimental ischemic stroke. J Pharmacol Exp Ther 332: 1006–1012. 10.1124/jpet.109.160937 19952306 PMC2835431

[19] Chan GC, Fish JE, Mawji IA, Leung DD, Rachlis AC, Marsden PA. (2005) Epigenetic basis for the transcriptional hyporesponsiveness of the human inducible nitric oxide synthase gene in vascular endothelial cells. J Immunol 175: 3846–3861. 16148131 10.4049/jimmunol.175.6.3846

[20] Wang YS, Chou WW, Chen KC, Cheng HY, Lin RT, Juo SH. (2012) MicroRNA-152 mediates DNMT1-regulated DNA methylation in the estrogen receptor alpha gene. PLoS One 7: e30635 10.1371/journal.pone.0030635 22295098 PMC3266286

[21] Baccarelli A, Wright R, Bollati V, Litonjua A, Zanobetti A, Tarantini L, et al (2010) Ischemic heart disease and stroke in relation to blood DNA methylation. Epidemiology 21: 819–828. 10.1097/EDE.0b013e3181f20457 20805753 PMC3690659

[22] Kim JM, Stewart R, Park MS, Kang HJ, Kim SW, Shin IS, et al (2012) Associations of BDNF genotype and promoter methylation with acute and long-term stroke outcomes in an East Asian cohort. PLoS One 7: e51280 10.1371/journal.pone.0051280 23240009 PMC3519835

[23] Lin RT, Hsi E, Lin HF, Liao YC, Wang YS, Juo SH. (2014) LINE-1 methylation is associated with an increased risk of ischemic stroke in men. Curr Neurovasc Res 11: 4–9. 24295503 10.2174/1567202610666131202145530

[24] Dubal DB, Kashon ML, Pettigrew LC, Ren JM, Finklestein SP, Rau SW, et al (1998) Estradiol protects against ischemic injury. J Cereb Blood Flow Metab 18: 1253–1258. 9809515 10.1097/00004647-199811000-00012

[25] Wise PM, Dubal DB (2000) Estradiol protects against ischemic brain injury in middle-aged rats. Biol Reprod 63: 982–985. 10993817 10.1095/biolreprod63.4.982

[26] Westberry JM, Trout AL, Wilson ME (2010) Epigenetic regulation of estrogen receptor alpha gene expression in the mouse cortex during early postnatal development. Endocrinology 151: 731–740. 10.1210/en.2009-0955 19966177 PMC2817618

[27] Chang PY, Chen YJ, Chang FH, Lu J, Huang WH, Yang TC, et al (2013) Aspirin protects human coronary artery endothelial cells against atherogenic electronegative LDL via an epigenetic mechanism: a novel cytoprotective role of aspirin in acute myocardial infarction. Cardiovasc Res 99: 137–145. 10.1093/cvr/cvt062 23519265

[28] Pereira MA, Tao L, Wang W, Li Y, Umar A, Steele VE, et al (2004) Modulation by celecoxib and difluoromethylornithine of the methylation of DNA and the estrogen receptor-alpha gene in rat colon tumors. Carcinogenesis 25: 1917–1923. 15205357 10.1093/carcin/bgh209

[29] Lodder J, Boiten J (1993) Incidence, natural history, and risk factors in lacunar infarction. Adv Neurol 62: 213–227. 8517211

[30] Alkayed NJ, Harukuni I, Kimes AS, London ED, Traystman RJ, Hurn PD. (1998) Gender-linked brain injury in experimental stroke. Stroke 29: 159–165; discussion 166. 9445346 10.1161/01.str.29.1.159

[31] Hall ED, Pazara KE, Linseman KL (1991) Sex differences in postischemic neuronal necrosis in gerbils. J Cereb Blood Flow Metab 11: 292–298. 1997500 10.1038/jcbfm.1991.61

[32] Turtzo LC, McCullough LD (2010) Sex-specific responses to stroke. Future Neurol 5: 47–59. 20190872 10.2217/fnl.09.66 PMC2827821

[33] Tian Y, Stamova B, Jickling GC, Liu D, Ander BP, Bushnell C, et al (2012) Effects of gender on gene expression in the blood of ischemic stroke patients. J Cereb Blood Flow Metab 32: 780–791. 10.1038/jcbfm.2011.179 22167233 PMC3345909

[34] Fuso A, Ferraguti G, Grandoni F, Ruggeri R, Scarpa S, Strom R, et al (2010) Early demethylation of non-CpG, CpC-rich, elements in the myogenin 5'-flanking region: a priming effect on the spreading of active demethylation. Cell Cycle 9: 3965–3976. 20935518 10.4161/cc.9.19.13193 PMC3047754

[35] Fuso A, Ferraguti G, Scarpa S, Ferrer I, Lucarelli M (2015) Disclosing bias in bisulfite assay: MethPrimers underestimate high DNA methylation. PLoS One 10: e0118318 10.1371/journal.pone.0118318 25692551 PMC4333220

