# Supplementary material for: Demethylation of Circulating Estrogen Receptor Alpha Gene in Cerebral Ischemic Stroke
Source: PLoS One. 2015 Sep 30;10(9):e0139608. doi: 10.1371/journal.pone.0139608 (PMC4589317; doi:10.1371/journal.pone.0139608)
Supplement: S1 Table — (DOCX) [file pone.0139608.s001.docx]

**S1 table.** Primers for Estrogen receptor 1(ESR1) methylation

| PCR primers for ESR1 |
| --- |
| Forward: 5’-GGGAGAGGTGTATTTGGATAGTAGTA-3’  Reverse: 5’-Biotin-ATTAAATAAAAAAAAACCCCCCAAAC-3’ |
| Sequence primer for ESR1 |
| 5’-GTGTATTTGGATAGTAGTAAG-3’ |
