# Supplementary material for: Demethylation of Circulating Estrogen Receptor Alpha Gene in Cerebral Ischemic Stroke
Source: PLoS One. 2015 Sep 30;10(9):e0139608. doi: 10.1371/journal.pone.0139608 (PMC4589317; doi:10.1371/journal.pone.0139608)
Supplement: S2 Table — (DOCX) [file pone.0139608.s002.docx]

**S2 table.** Stratification analysis for estrogen receptor α promoter methylation status in large-artery atherosclerosis and cardio-embolic stroke subtypes by sex

|  |  | Men |  |  | Women |  |
| --- | --- | --- | --- | --- | --- | --- |
|  | Controls n=101 | Stroke n=26 | Adjusted *p* value | Controls n=116 | Stroke n=33 | Adjusted *p* value |
| Methylation % | Mean±SD | Mean ± SD |  | Mean±SD | Mean ± SD |  |
| Site5 | 4.02±1.25 | 3.82±1.56 | 0.736 | 4.62±2.59 | 3.34±1.58 | **0.024** |
| Site9 | 2.79±0.93 | 2.56±1.05 | 0.258 | 3.05±1.22 | 2.27±1.05 | **0.014** |
| Site12 | 4.20±1.52 | 3.86±1.88 | 0.377 | 4.57±1.90 | 3.39±2.00 | **0.023** |
| Site13 | 3.04±1.91 | 2.71±1.06 | 0.286 | 3.06±1.27 | 2.29±1.16 | 0.061 |
| Site14 | 7.64±3.59 | 7.11±2.41 | 0.367 | 8.08±3.64 | 6.34±2.52 | **0.022** |

Adjusted p value was adjusted for age, hypertension, diabetes, hypercholesterolemia, and smoking
